# Added value of the measles-rubella supplementary immunization activity in reaching unvaccinated and under-vaccinated children, a cross-sectional study in five Indian districts, 2018–20

**DOI:** 10.1016/j.vaccine.2022.11.010

**Published:** 2023-01-09

**Authors:** C. Prosperi, J.W.V. Thangaraj, A.Z. Hasan, M.S. Kumar, S. Truelove, V.S. Kumar, A.K. Winter, A.K. Bansal, S.L. Chauhan, G.S. Grover, A.K. Jain, R.N. Kulkarni, S.K. Sharma, B. Soman, I.K. Chaaithanya, S. Kharwal, S.K. Mishra, N.R. Salvi, N.P. Sharma, S. Sharma, A. Varghese, R. Sabarinathan, A. Duraiswamy, D.S. Rani, K. Kanagasabai, A. Lachyan, P. Gawali, M. Kapoor, S.K. Chonker, F.T. Cutts, L. Sangal, S.M. Mehendale, G.N. Sapkal, N. Gupta, K. Hayford, W.J. Moss, M.V. Murhekar

**Affiliations:** a International Vaccine Access Center, Department of International Health, Johns Hopkins Bloomberg School of Public Health, Baltimore, MD, USA; b Indian Council of Medical Research (ICMR)-National Institute of Epidemiology, Chennai, India; c ICMR-National JALMA Institute for Leprosy & Other Mycobacterial Diseases, Agra, India; d ICMR- National Institute for Research in Reproductive and Child Health (NIRRCH), Mumbai, India; e Directorate of Health Services, Government of Punjab, Chandigarh, India; f ICMR-National Institute of Pathology, New Delhi, India; g ICMR-Regional Medical Research Centre, NE Region, Dibrugarh, India; h Achutha Menon Centre for Health Science Studies, Sree Chitra Tirunal Institute for Medical Sciences and Technology, Trivandrum, Kerala, India; i Department of Health Research, Model Rural Health Research Unit-Dahanu, Maharashtra, India; j Department of Health Research, Model Rural Health Research Unit-Hoshiarpur, Punjab, India; k Department of Health Research, Model Rural Health Research Unit-Chabua, Assam, India; l Department of Health Research, Model Rural Health Research Unit-Kanpur, Uttar Pradesh, India; m Department of Infectious Disease Epidemiology, London School of Hygiene and Tropical Medicine, London, UK; n World Health Organization, Southeast Asia Region Office, New Delhi, India; o PD Hinduja Hospital and Medical Research Centre, Mumbai, India; p ICMR-National Institute of Virology, Pune, India; q Division of Epidemiology and Communicable Diseases, Indian Council of Medical Research, New Delhi, India; r Department of Epidemiology, Johns Hopkins Bloomberg School of Public Health, Baltimore, MD, USA

**Keywords:** Measles, Routine immunization, Supplementary immunization activities, Coverage, Surveys

## Abstract

**Introduction:**

Supplementary immunization activities (SIAs) aim to interrupt measles transmission by reaching susceptible children, including children who have not received the recommended two routine doses of MCV before the SIA. However, both strategies may miss the same children if vaccine doses are highly correlated. How well SIAs reach children missed by routine immunization is a key metric in assessing the added value of SIAs.

**Methods:**

Children aged 9 months to younger than 5 years were enrolled in cross-sectional household serosurveys conducted in five districts in India following the 2017–2019 measles-rubella (MR) SIA. History of measles containing vaccine (MCV) through routine services or SIA was obtained from documents and verbal recall. Receipt of a first or second MCV dose during the SIA was categorized as “added value” of the SIA in reaching un- and under-vaccinated children.

**Results:**

A total of 1,675 children were enrolled in these post-SIA surveys. The percentage of children receiving a 1st or 2nd dose through the SIA ranged from 12.8% in Thiruvananthapuram District to 48.6% in Dibrugarh District. Although the number of zero-dose children prior to the SIA was small in most sites, the proportion reached by the SIA ranged from 45.8% in Thiruvananthapuram District to 94.9% in Dibrugarh District. Fewer than 7% of children remained measles zero-dose after the MR SIA (range: 1.1–6.4%) compared to up to 28% before the SIA (range: 7.3–28.1%).

**Discussion:**

We demonstrated the MR SIA provided considerable added value in terms of measles vaccination coverage, although there was variability across districts due to differences in routine and SIA coverage, and which children were reached by the SIA. Metrics evaluating the added value of an SIA can help to inform the design of vaccination strategies to better reach zero-dose or undervaccinated children.

## 1 Introduction

India made significant progress in increasing coverage with measles-containing vaccines (MCVs) over the past two decades and reduced inequalities within and across states [1]. As a consequence, estimated measles mortality declined, from 3.3 per 1,000 livebirths in 2000 to 0.3 per 1,000 livebirths in 2015 [2]. But despite reductions in measles incidence and mortality, India has one of the highest numbers of measles cases globally because of its large population size and suboptimal routine coverage in some states [3], [4]. With disruptions to immunization services as a result of the SARS-CoV-2 pandemic, there are concerns that years of progress in measles control and elimination may be stalled or reversed [5], [6].

As part of India’s commitment to measles elimination and rubella control, the government launched a measles-rubella (MR) SIA in 2017 to reduce measles transmission and introduce rubella vaccine in the national immunization program, targeting all children aged 9 months to less than 15 years regardless of prior vaccination status [7]. For most children, who had not received rubella-containing vaccine through the private sector [8], this SIA provided the first vaccine dose against rubella virus, which can cause congenital rubella syndrome (CRS) in children born to susceptible women if infected early in pregnancy [9]. Approximately 40,000 cases of CRS were estimated to occur in India in 2010, the highest number of cases globally [10]. The MR SIA was implemented in phases, beginning in 2017, and continued for approximately-three years, vaccinating children in schools, health facilities, and outreach locations. Following the SIA, the MR vaccine was introduced into the Universal Immunisation Programme as a replacement for the monovalent measles vaccine, given at 9 to 12 months and 16 to 24 months of age [7].

The use of SIAs as a key strategy for measles elimination was pioneered in the Americas, where interruption of measles transmission and maintenance of low or absent transmission was monitored closely using high-quality measles surveillance [11]. Elsewhere, SIAs have become a regular component of measles control activities with SIA coverage being used as a proxy measure of success. To have greatest impact, SIAs need to reach measles zero-dose and under-vaccinated children missed by the routine immunization program, ideally before they acquire measles [12], [13]. However, both routine and supplementary strategies may miss the same children if vaccine doses are highly correlated, i.e., if children receiving a supplementary dose are a subset of those who received a routine dose [14]. How well SIAs reach children missed by the routine immunization program is a key metric in assessing their added value. In this paper, we are defining “added value” as receipt of the first or second dose of MCV through the SIA, while recognizing that this is an incomplete proxy for overall added value in the absence of information on natural immunity [15]. Measuring these indicators of reaching under-vaccinated children as part of post-SIA surveys has recently been emphasized, such as the proportion of children receiving the supplementary dose stratified by the number of routine doses received prior to the SIA [12], [16]. We conducted cross-sectional serosurveys in five districts in India following the 2017–2019 MR SIA and describe the added value of the SIA in reaching MCV unvaccinated and under-vaccinated children.

## 2 Methods

### 2.1 Survey setting

The study was conducted from March 2018 to March 2020 to estimate measles and rubella seroprevalence among children in two age groups (9 months to younger than 5 years, 5 to younger than 15 years) and to evaluate the impact of the nationwide MR SIA conducted from 2017 to 2019. This analysis was restricted to children enrolled in cross-sectional serosurveys conducted following the MR SIA in Dibrugarh District, Assam; Hoshiarpur District, Punjab; Palghar District, Maharashtra; Kanpur Nagar District, Uttar Pradesh; and Thiruvananthapuram District, Kerala (Supplementary Fig. 1). These districts varied in characteristics that impact vaccination coverage and the effectiveness of the SIA in reaching under-vaccinated children, such as rural and urban differences, health care quality, adult literacy and educational attainment, and access to private healthcare (Supplementary Table 1). For example, Dibrugarh District is primarily rural with special subpopulations such as those living on tea garden estates. The percent of adult females with 10 or more years of school ranged from 34 % in Dibrugarh District, Assam to 83 % in Thiruvananthapuram District, Kerala, based on data from the National Family Health Survey (2019–2020) [17]. MCV1 coverage among 12–23 month old children according to card or verbal recall in surveys conducted in 2019–2020 ranged from 79 % in Kanpur Nagar District to over 90 % in the remaining four districts [17].

### 2.2 Survey methods

In each district, a separate three-stage cluster sample survey was conducted. At the first stage, thirty clusters (villages or wards) were selected using probability proportional to population size systematic sampling based on the 2011 nationwide census. At the second stage, one Census Enumeration Block (CEB) was randomly selected from the list of CEBs in each selected cluster. A CEB is a well-defined area with approximately 120 to 150 households and approximately 600–750 persons used for census purposes [18]. All individuals in the selected CEB (including those who slept in the houses the prior night) were enumerated by trained survey teams using a tablet-based form. If the cluster was too large to complete enumeration within two days based on the approximate number of households, it was segmented and one segment was randomly selected for enumeration (see Supplementary Methods for more information). At the final stage, thirteen children were randomly selected from each of the two age strata using a tablet-based application, for a total of 26 children per cluster. Prior to enrollment, written informed consent was obtained from the parents/legal guardian and assent was obtained from children aged 7 to younger than 15 years. Three of the thirty selected clusters in Dibrugarh, Assam could not be completed due to the COVID-19 pandemic. Sample size was estimated for the overall serosurvey conducted in these districts, assuming rubella seroprevalence of 50 % among both the younger and older age groups, with an absolute precision of 10 %, a design effect of 2, and a 95 % confidence level [15].

After obtaining consent, survey staff collected socio-demographic and vaccination history data from the parents or caregiver of the child using a standardized, tablet-based questionnaire. Recall of routine MCV receipt was asked from all caregivers of children aged 9 months to younger than 5 years prior to reviewing the vaccination card. Documented receipt of MCV was recorded for those with available routine vaccination cards; health facilities were not visited for those lacking home-based records. Information on receipt of an MR vaccine supplementary dose was collected for all enrolled children based on an MR SIA card, where available, or caregiver recall. For recall of both routine and supplementary doses, standardized language was used to describe the vaccines to the caregiver, such as how the vaccine is administered (e.g., shot in upper arm) and details about the SIA (e.g., “occurring between X-Y dates”, “may have been given at a local school or health center”).

The surveys were led by the Indian Council of Medical Research (ICMR) National Institute of Epidemiology (NIE), Chennai, and locally implemented by researchers at the Model Rural Health Research Unit (MRHRU) or other research institute in each district.

### 2.3 Analysis

Vaccination coverage analyses were restricted to children 9 months to younger than 5 years at the time of the SIA as routine vaccination data were only collected for this age group. Crude routine and SIA coverage (i.e., not accounting for timeliness of vaccination) were estimated based on home-based (vaccine card) record documentation only, recall only, and a composite measure was derived using either source of information. Children were categorized based on the number of doses received and how each dose was administered (routine or supplementary). The sensitivity of routine recall was estimated as the proportion of children with card documented evidence of vaccination whose caregiver recalled receipt. Routine vaccine coverage was estimated based on receipt at time of the MR SIA. Any documented routine doses administered after the start of the SIA in that district were excluded from analyses. We did not record the date when the child received the supplementary dose, so the SIA start date for the district where the child resided was used to exclude post-SIA routine doses. For verbal recall of routine vaccination, some doses may have been administered after the SIA but were counted towards the child’s status at the time of the SIA in the absence of documentation of the vaccination date. Children with unknown vaccination recall status were treated as not having received a dose, although sensitivity analyses were conducted treating these children as vaccinated and then repeated after excluding these children.

Children who received their first or second MCV dose during the SIA were categorized as receiving “added value” from the SIA. Analyses were conducted for each district survey and stratified by individual and household characteristics such as gender, maternal education, setting (urban or rural), housing materials, and type of facility where the child received vaccines (private or public). Site-specific comparisons between added value of the SIA and individual or household characteristics were made using multinomial logistic regression, adjusted for age at the SIA. Select results were presented as all-site summaries, calculated as simple averages of the site-specific survey data.

Coverage estimates with 95 % Wald confidence intervals accounted for sampling weights, including the probability of selection of the village or ward from the district, selection of the CEB, segmentation within the selected CEB if applicable, the probability of selecting the child from all enumerated children in the cluster, and corrected for non-response. All analyses were performed using SAS statistical software (version 9.4; SAS, Cary, NC, USA) and R (version 3.4.4, Vienna, Austria).

### 2.4 Ethics

This study was reviewed and approval by the Institutional Human Ethics Committee of the ICMR National Institute of Epidemiology, Chennai, the Institutional Review Board at the Johns Hopkins Bloomberg School of Public Health, and locally by each institution’s ethical review committee.

## 3 Results

### 3.1 Characteristics of survey respondents

One thousand six hundred and seventy-five children aged 9 months to younger than 5 years at the time of the MR SIA were enrolled in surveys across five districts in India conducted between three to 16 months after the MR SIA (Table 1, Supplementary Fig. 2). Approximately 88 % of those selected for the survey agreed to participate, with similar enrollment rates in all districts. Across all districts, most (53.0 %) mothers had attained middle to higher secondary education, with the highest level of education observed in Thiruvananthapuram District. Most houses were constructed with permanent (54.8 %) or semi-permanent (22.4 %) materials. Survey clusters in Hoshiarpur and Dibrugarh Districts were mostly rural, whereas those in Kanpur Nagar District tended to be urban; clusters in other districts were evenly distributed. Thirty-six percent of children enrolled in Dibrugarh District lived in a survey cluster containing a tea estate. Few children received routine vaccines from private facilities (3.2 % in Dibrugarh District up to 14.5 % in Thiruvananthapuram District).

**Table 1:** Individual and household characteristics of survey sample, children 9 months to younger than 5 years of age at the time of the measles and rubella supplementary immunization activity (SIA).

	Thiruvananthapuram District, Kerala	Kanpur Nagar District, Uttar Pradesh	Palghar District, Maharashtra	Hoshiarpur District, Punjab	Dibrugarh District, Assam	All Sites
	n (%)	n (%)	n (%)	n (%)	n (%)	n (%)
**Number selected**	**387**	**388**	**387**	**390**	**351**^a^	**1903**
**Total enrolled (% of those selected)**	340 (88.1)	341 (88.8)	336 (87.7)	346 (88.7)	312 (90.2)	1675 (88.7)
**Approximate time between SIA and survey^b^**	10–16 m	3–6 m	3–5 m	13–16 m	12–14 m	–
*Participant Characteristics*						
Female	165 (48.5)	166 (48.7)	178 (53.0)	171 (49.4)	148 (47.4)	828 (50.6)
Child’s mother was respondent	248 (72.9)	287 (84.2)	267 (79.5)	279 (80.6)	211 (67.6)	1292 (77.1)
Age at survey (years), median (IQR)	4.3 (3.1, 5.4)	3.6 (2.5, 4.5)	3.1 (2.1, 4.2)	4.6 (3.7, 5.4)	4.5 (3.3, 5.3)	4.0 (2.9, 5.0)
*Household Characteristics*						
Maternal education level						
Graduate or above	142 (41.9)	82 (24.2)	49 (14.6)	74 (21.4)	23 (7.5)	370 (22.2)
Middle to higher secondary	179 (52.6)	156 (46.0)	176 (52.4)	224 (64.9)	149 (48.4)	884 (53.0)
Primary	14 (4.1)	46 (13.6)	37 (11.0)	23 (6.7)	112 (36.4)	232 (13.9)
Illiterate	4 (1.2)	55 (16.2)	74 (22.0)	24 (7.0)	24 (7.8)	181 (10.9)
Household materials						
Permanent	221 (65.0)	195 (57.2)	149 (44.3)	304 (88.9)	47 (15.1)	916 (54.8)
Semi-permanent	86 (25.3)	106 (31.1)	121 (36.0)	21 (6.1)	41 (13.1)	375 (22.4)
Non-permanent^c^	33 (9.7)	40 (11.7)	66 (19.6)	17 (5.0)	224 (71.8)	380 (22.7)
Immunization facility type						
Public	290 (85.5)	294 (88.6)	306 (91.1)	325 (95.3)	302 (96.8)	1517 (91.4)
Private	49 (14.5)	38 (11.4)	30 (8.9)	16 (4.7)	10 (3.2)	143 (8.6)
Setting						
Urban non-slum	109 (32.1)	92 (27.0)	91 (27.1)	108 (31.3)	37 (11.9)	437 (26.1)
Urban slum	56 (16.5)	130 (38.1)	82 (24.4)	12 (3.5)	30 (9.6)	310 (18.5)
Rural	175 (51.5)	119 (34.9)	163 (48.5)	225 (65.2)	245 (78.5)	927 (55.4)

a. Three selected clusters in Dibrugarh, Assam could not be completed due to the COVID-19 pandemic.

b. Approximate time between SIA and survey based on approximate district-level SIA start date (minimum) and stop date (maximum) and the date the serosurvey was started**.**

c. Houses in Dibrugarh District, Assam are commonly built with non-permanent materials given the local context and environment. In this setting the housing materials may be less reflective of socioeconomic status as they may be in other settings.

### 3.2 Data sources and quality

Availability of routine vaccination cards ranged from 51.6 % of children in Kanpur Nagar District to 83.8 % in Thiruvananthapuram District (Table 1). Fewer than half of children enrolled in Kanpur Nagar (47.6 %) District and only 10.3 % of children in Thiruvananthapuram District had an SIA card available. In Thiruvananthapuram District this was primarily due to limited availability and distribution of the SIA cards to health facilities (personal communication). SIA card availability in the other three districts ranged from 61.7 % to 76.1 %.

In Kanpur Nagar and Palghar Districts, over 35 % of children had evidence of routine vaccination by recall only. Sensitivity of maternal recall was greater than 90 % in Thiruvananthapuram, Kanpur Nagar, Palghar, and Hoshiarpur Districts (Supplementary Table 2). However, in Dibrugarh District most caregivers of children with documented evidence were uncertain when asked to recall their child’s vaccination history, with only 26.1 % accurately recalling MCV receipt and 69.6% indicating vaccination status was unknown. On average across the districts, fifty percent of caregivers whose child had a card available that showed no evidence of MCV stated that the child received the vaccine when asked to recall. This discrepancy may have been due to incorrect recall by the caregiver or issues with documentation at time of vaccination, however the number of applicable children is small (Supplementary Table 2). Twenty-percent of children lacked card or positive recall evidence on routine doses in Hoshiarpur (MCV2) and Dibrugarh (both doses) Districts (Table 2); children for whom the respondent was not the mother were more likely to be uncertain of vaccination status compared to children whose mother was the respondent (Supplementary Table 3).

**Table 2:** Routine MCV coverage among children aged 9 months to below 5 years of age at the time of the MR SIA and coverage of the SIA.

	Thiruvananthapuram District, Kerala	Kanpur Nagar District, Uttar Pradesh	Palghar District, Maharashtra	Hoshiarpur District, Punjab	Dibrugarh District, Assam
	% (95 % CI)	% (95 % CI)	% (95 % CI)	% (95 % CI)	% (95 % CI)
*Card availability^a^*					
Routine	285 (83.8)	176 (51.6)	224 (66.7)	259 (74.9)	233 (74.7)
SIA	34 (10.3)	161 (47.6)	255 (76.1)	211 (61.7)	201 (65.5)
*Routine immunization*					
MCV1					
Documented	81.6 (76.3, 85.9)	41.9 (33.3, 51)	55.1 (45.4, 64.4)	68.2 (59.7, 75.7)	68.2 (59.5, 75.8)
Positive Recall only	11.1 (7.6, 15.8)	45.9 (38.6, 53.4)	36.2 (27.8, 45.6)	18.8 (11.2, 29.8)	3.6 (1.4, 8.6)
Documented + positive recall	92.7 (87.8, 95.7)	87.8 (80.9, 92.5)	91.3 (85.3, 95)	87.0 (81.5, 91.1)	71.8 (62.2, 79.8)
No evidence^b^	3.3 (1.8, 6.1)	8.2 (4.5, 14.7)	6.6 (3.9, 11.1)	4.1 (2.2, 7.7)	8.5 (5.3, 13.6)
Unknown^c^	4.0 (1.9, 8.1)	3.9 (2.3, 6.8)	2.1 (0.7, 6.3)	8.8 (5.3, 14.4)	19.6 (12.9, 28.8)
MCV2					
Documented	68.8 (64, 73.3)	28.2 (21.1, 36.5)	35.0 (28, 42.8)	52.5 (44.4, 60.5)	42.6 (34.2, 51.6)
Positive recall only	9.9 (6.7, 14.5)	36.6 (29, 44.9)	37.1 (27, 48.5)	4.0 (2.2, 7.2)	3.1 (1.1, 8.2)
Documented + positive recall	78.8 (74.3, 82.6)	64.7 (54.1, 74.1)	72.1 (63, 79.7)	56.5 (47.8, 64.8)	45.7 (36.4, 55.3)
No evidence^d^	16.9 (13.6, 20.9)	30.7 (22.2, 40.9)	23.5 (16.0, 33.1)	22.2 (17.7, 27.4)	32.4 (25.3, 40.5)
Unknown^e^	4.3 (2.1, 8.5)	4.5 (2.8, 7.2)	4.4 (2.0, 9.3)	21.3 (14.0, 31.0)	21.9 (15.6, 29.8)
Supplementary immunization SIA					
Documented	8.7 (5.5, 13.6)	44.8 (33.2, 56.9)	69.1 (60.2, 76.8)	58.6 (48.8, 67.8)	62.4 (49.7, 73.6)
Positive recall only	70.9 (59, 80.5)	28.4 (21, 37.1)	20.5 (14.4, 28.3)	24.4 (16.6, 34.4)	28.1 (18.2, 40.8)
Documented + positive recall	79.6 (65.8, 88.8)	73.2 (63.3, 81.2)	89.6 (85.3, 92.8)	83.1 (74.3, 89.3)	90.5 (83.8, 94.7)
No evidence^f^	16.3 (8.7, 28.4)	25.3 (18.1, 34.2)	10.2 (7.0, 14.7)	15.0 (10.1, 21.7)	8.5 (4.7, 14.9)
Unknown^g^	4.1 (1.6, 9.9)	1.5 (0.3, 6.8)	0.2 (0.02, 1.3)	1.9 (0.5, 6.5)	1.0 (0.3, 3.6)
SIA Coverage by number of prior doses					
‘True’ zero-dose	50.6 (19.0, 81.8)	44.8 (21.6, 70.5)	83.1 (47.6, 96.4)	81.5 (47.7, 95.5)	94.0 (75.1, 98.8)
One	63.8 (41.5, 81.5)	71.8 (56.5, 83.2)	87.9 (75.6, 94.4)	83.1 (64.5, 93.0)	83.6 (70.5, 91.6)
Two	85.6 (73.8, 92.6)	78.5 (68.9, 85.7)	90.5 (83.6, 94.7)	87.6 (82.1, 91.7)	91.8 (80.4, 96.9)
Unknown	42.7 (13.7, 77.7)	53.3 (23.2, 81.2)	92.3 (52.3, 99.2)	54.3 (30.4, 76.4)	95.3 (84.5, 98.7)
SIA Coverage among MCV zero-dose children^h^	45.8 (22.5, 71.1)	47.5 (27.6, 68.2)	85.7 (57.9, 96.3)	63.0 (42.5, 79.7)	94.9 (87.9, 97.9)
SIA Coverage among children with a prior MCV dose from routine^h^	82.0 (69.2, 90.2)	76.7 (67.4, 84.0)	89.9 (85.5, 93.1)	86.1 (78.9, 91.0)	88.8 (81.7, 93.4)

Vaccination coverage estimates are survey weighted. Documented doses administered after the start of the SIA in the district were excluded from this analysis.

a. Card available and shown to interviewer. SIA card availability was lower in Thiruvananthapuram District due to limited availability and limited distribution at the time of the SIA. Unweighted estimates.

b. Mother/caregiver reported child did not receive MCV1 and card is either available but blank for MCV1 or not available.

c. No card available and mother/caregiver reported not knowing if child received MCV1.

d. Mother/caregiver reported child did not receive MCV, or reported child only received one dose of MCV, and card is either available but blank for MCV2 or not available.

e. No card available and mother/caregiver reported not knowing if child received MCV2, or reported child received MCV but number of doses unknown.

f. Mother/caregiver reported child did not receive SIA dose and card is either available but blank or not available.

g. No card available and mother/caregiver reported not knowing if child received SIA dose.

h. Restricted to children 9 m to less than 5 years (at time of the SIA), regardless of immunization card availability. Receipt of routine and SIA doses based on card documentation (irrespective of age at receipt of MCV) or positive recall. Children with unknown vaccination status were treated as unvaccinated. Number of zero-dose children before SIA: Kerala, N = 23; Uttar Pradesh, N = 37; Maharashtra, N = 30; Punjab, N = 44; Assam, N = 89.

### 3.3 Supplementary immunization activity coverage and added value of SIA

Routine MCV coverage at the time of SIA, defined as receipt by card or recall, varied from 71.8 % to 92.7 % for the first dose and 45.7 % to 78.8 % for the second dose by district, when those with “unknown” recall were classed as unvaccinated (Table 2). MR SIA coverage was below 80 % in Kanpur Nagar (73.2 %) and Thiruvananthapuram (79.6 %) Districts and only Dibrugarh District attained greater than 90 % SIA coverage. Most children in Palghar, Hoshiarpur, and Dibrugarh Districts had card documented evidence of MR SIA receipt (Table 2).

The proportion of measles zero-dose children who were reached by the SIA varied by district (Thiruvananthapuram: 45.8 % of 23 [95 % CI, 22.5, 71.1]; Kanpur Nagar: 47.5 % of 37 [95 % CI 27.6, 68.2]; Hoshiarpur: 63.0 % of 44 [95 % CI 42.5, 79.7]; Palghar: 85.7 % of 30 [95 % CI 57.9, 96.3]; Dibrugarh: 94.9 % of 89 [95 % CI 87.9, 97.9]) when treating unknown vaccination status as unvaccinated (Table 2). Dibrugarh District, with the lowest estimated MCV1 and MCV2 coverage prior to the SIA, had the highest proportion of measles zero-dose children reached by the SIA (94.9 %). SIA coverage among those children who had received a prior dose of MCV ranged from 76.7 % to 89.9 %. In Thiruvananthapuram, Kanpur Nagar, and Hoshiarpur districts, children who were measles zero-dose prior to the SIA were significantly less likely to have received the SIA dose compared to those with a prior measles dose (Supplementary Table 4). Measles zero-dose children in Dibrugarh District were more likely to have received the SIA dose compared to those with a prior dose, though the finding was not significant.

**Fig. 1:**
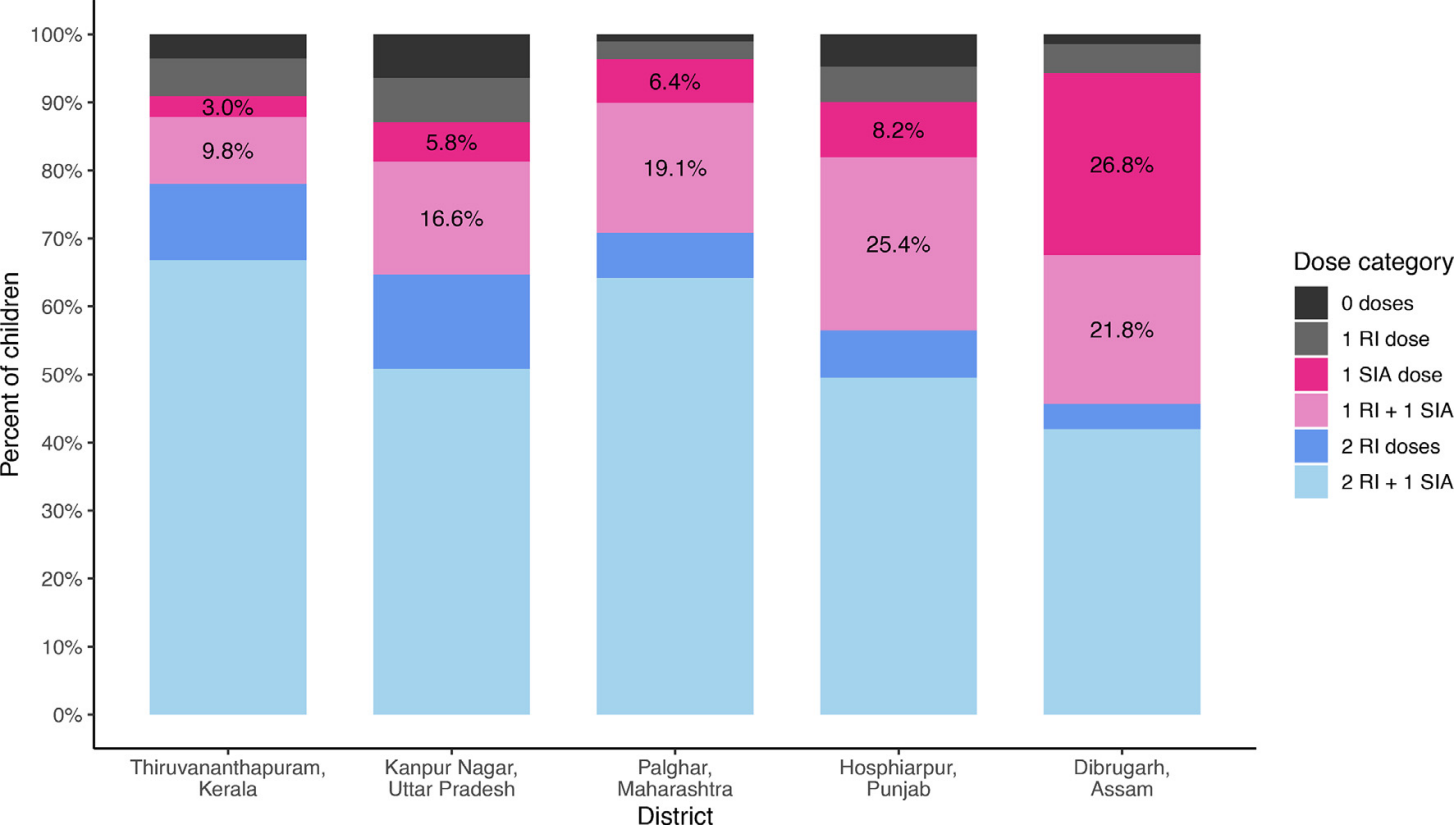
Receipt of measles-containing vaccine among children 9 months to younger than 5 years of age post-SIA by site and strategy, including documented or recall evidence of vaccination. Footnote: Bottom three bars reflect children who received at least one dose from the routine system; if respondent was uncertain vaccine was coded as not given. Pink bars reflect children who received added value from the SIA in terms of measles vaccine, either their first (dark pink) or second (light pink) dose of MCV. Dark gray bar at the top reflects the “zero MCV dose” children after SIA. Receipt for all doses is based on documented receipt plus positive recall. Percentages are survey weighted. (For interpretation of the references to colour in this figure legend, the reader is referred to the web version of this article.)

**Fig. 2:**
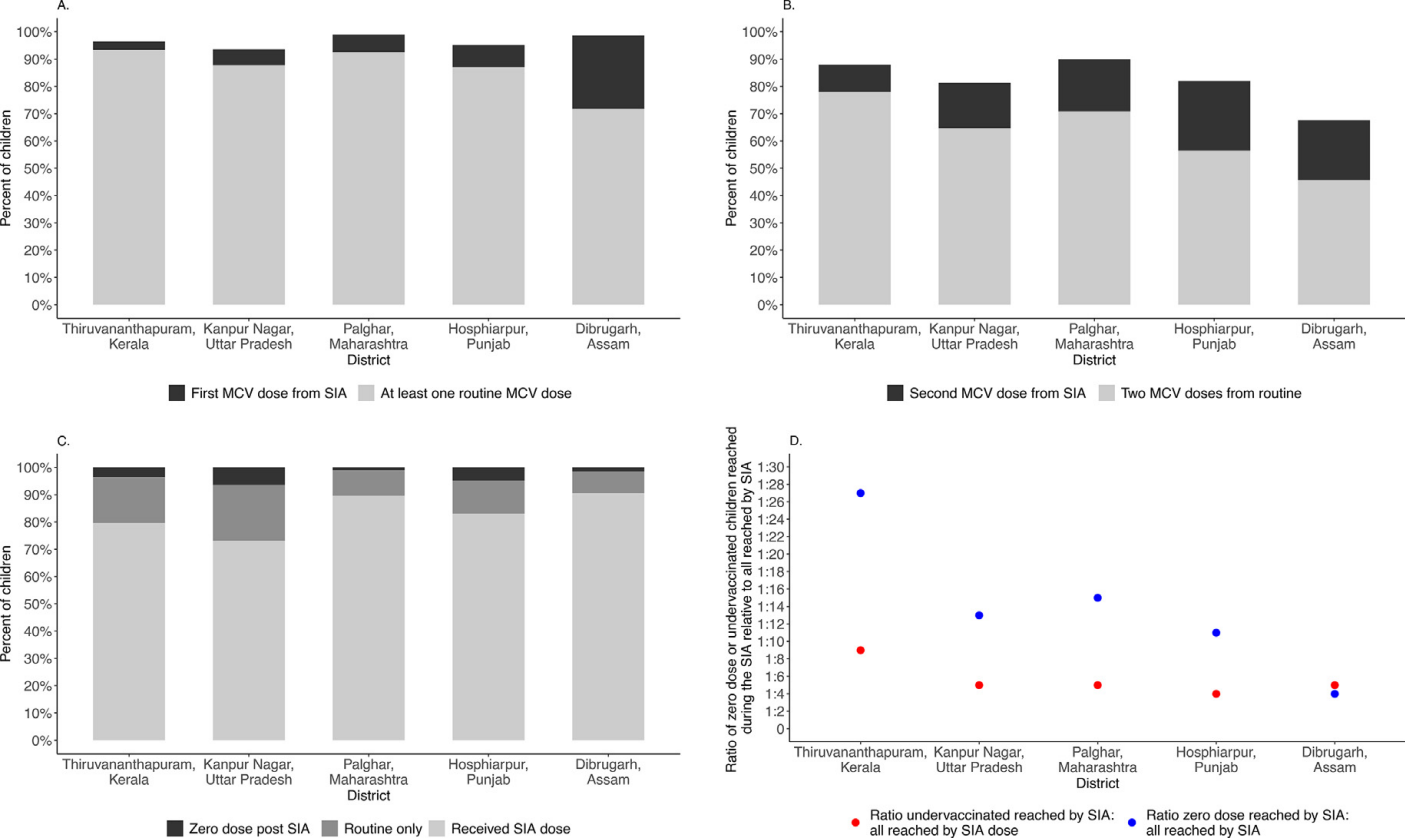
Impact of the supplementary immunization activity (SIA) on MCV receipt among children younger than 5 years of age by site. Footnote: A. Net increase in MCV1 receipt; B. Net increase in MCV2 receipt; C. Receipt of the MR SIA relative to those children with zero doses after the SIA; D. Ratio of zero dose or under-vaccinated children reached during the SIA relative to all reached by SIA. Restricted to children <5 years. Receipt defined based on card plus recall; if respondent was uncertain of vaccination status the vaccine was coded as not given. Weighted frequencies and percentages for each dose category. Ratio calculated as the weighted number receiving the SIA dose divided by the weighted number of children reached in the SIA who were previously zero dose or under-vaccinated (rounded up to the nearest whole number).

Although SIA coverage was high among surveyed zero-dose children in three of the five districts, the net increase in the MCV1 coverage after the SIA was less than 10 % in all districts except Dibrugarh, where more than one-quarter (26.8%) of children received their first MCV dose through the SIA when treating children with unknown vaccination status as unvaccinated (Fig. 1 [dark pink], Fig. 2A, Supplementary Table 5A). There was substantially greater net increase in the percentage of children who received their second MCV dose during the SIA – from 16.6 % to 25.4 % across districts except for Thiruvananthapuram (9.8%) (Fig. 1 [light pink], Fig. 2B, Supplementary Table 5A). Overall, the percentage of children receiving added value from the SIA, defined as a first or second dose of MCV, ranged from 12.8 % in Thiruvananthapuram District to 48.6 % in Dibrugarh District (remaining districts: Kanpur Nagar, 22.4 %; Palghar, 25.4 %; Hoshiarpur, 33.5 %; Fig. 1 [pink bars], Supplementary Table 6) when classifying children with unknown vaccination status as unvaccinated.

When treating children with unknown routine MCV vaccination status as vaccinated, the percentage of children receiving their first or second dose of MCV decreased in Hoshiarpur District (from 33.5 % to 18.8 %) and Dibrugarh District (from 48.6 % to 28.4 %), but remained similar in the other sites where there were fewer children with unknown vaccination status (Supplementary Table 6). Similar findings were observed after excluding children with uncertain data on routine MCV doses (i.e., no card available and recall unknown) (Supplementary Table 6). However, the proportion of children receiving their first or second dose remained the highest in Dibrugarh District and lowest in Thiruvananthapuram District. Approximately 5 % of children in this analysis were within the timely receipt window for the first routine dose of MCV (aged 9–12 months at SIA) and 15 % of children within the timely receipt window for the second routine dose of MCV (aged 16–24 months at SIA). Restricting to children above 12 months of age, the percent of children receiving their first MCV dose from the SIA remained similar to the primary analysis with all children (range 2.9 % to 24.1 %) (Supplementary Fig. 3). When restricting to children above 24 months, the proportion who received their second dose from the SIA decreased relative to the analysis with all children (Supplementary Fig. 4). However, the proportion receiving their first dose from the SIA remained similar, as did the trends across the sites.

Fewer than 6 % of children remained measles zero-dose after the MR SIA (Fig. 1 [darkest gray], Fig. 2C, Supplementary Table 5A). The number of children remaining zero-dose ranged from 3 (1 %) in Palghar District, Maharashtra to 20 (6 %) in Kanpur Nagar District, Uttar Pradesh. The mean age of zero-dose children at time of SIA across all districts was 3.2 years. Caregivers of these children were less likely to be aware of the SIA (44 %) relative to other caregivers whose children had at least one dose (93 %), and 29 % of these mothers were illiterate, compared to 10 % of other caregivers (data not tabulated). Assuming 84 % of children receiving a single dose and 97 % of those receiving two or more doses are protected against measles virus [19], [20], [21], and ignoring potential natural immunity, we estimate that more than 89 % of children in all districts were protected by vaccination against measles post-SIA (range 89 % to 98 %; data not shown).

Using MR SIA coverage and proportion of zero-dose children reached by the SIA, in Dibrugarh District one measles zero-dose child was vaccinated for every-four children vaccinated during the SIA when treating unknown vaccination status as unvaccinated (Fig. 2D, Supplementary Table 5A). In contrast, the ratio of zero-dose children reached by the SIA relative to all children reached by the SIA ranged from more than 1:11 in Hoshiarpur District, Punjab to 1:27 in Thiruvananthapuram, District, Kerala. These ratios increased in all districts when treating children with unknown vaccination status as vaccinated (Supplementary Table 5B). The SIA provided a second dose for up to one in every-nine children vaccinated in the SIA (range 1:4 to 1:9; Supplementary Table 5A).

### 3.4 Added value of the MR SIA by individual and household characteristics

**Fig. 3:**
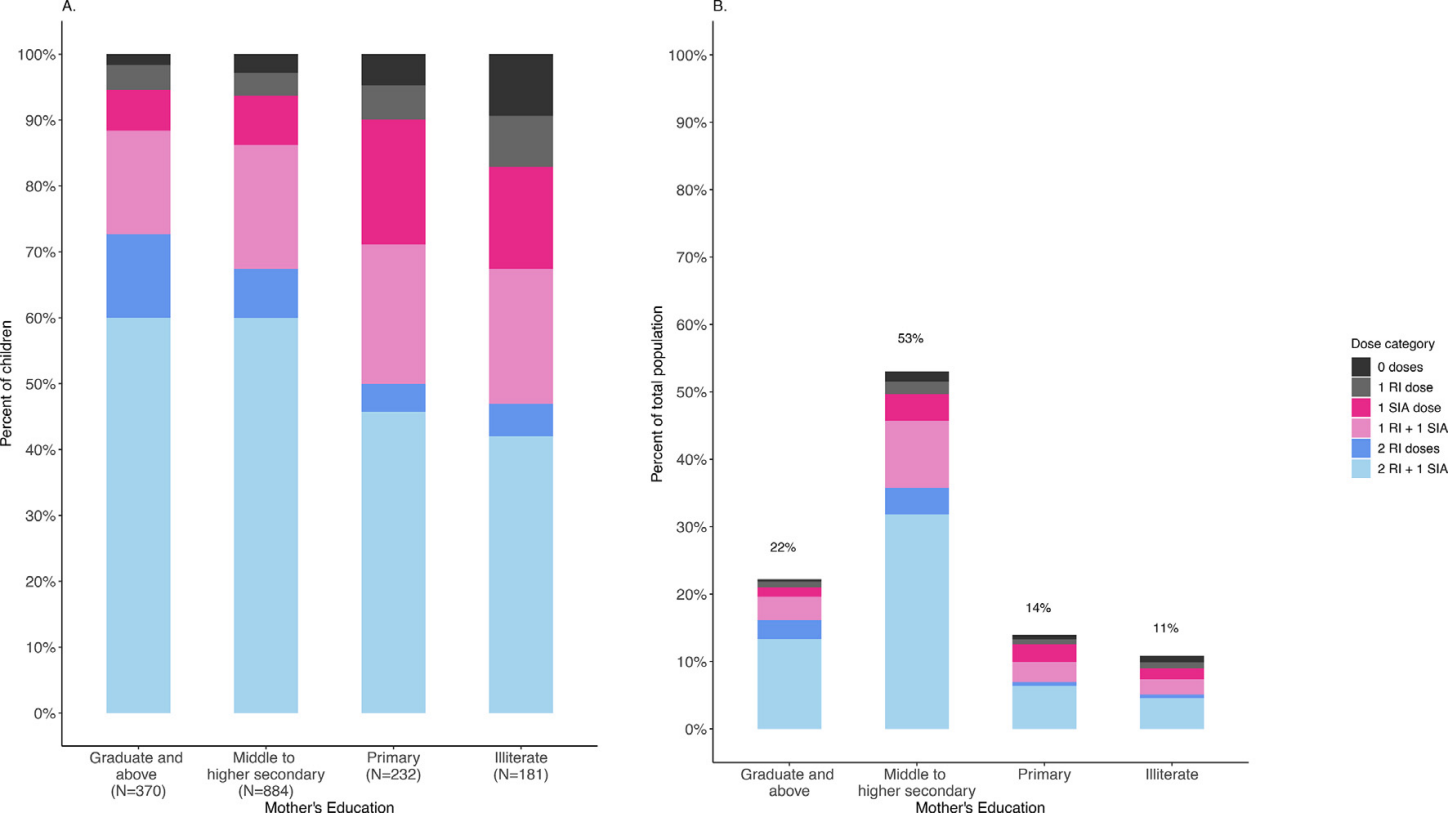
Routine MCV coverage among children aged 9 months to below 5 years of age at the time of the MR SIA and coverage of the SIA by maternal educational level, all districts combined. Footnote: Receipt for all doses is based on documented receipt plus recall. Left-hand figure is similar to Fig. 1, stratified by maternal education rather than district; numbers in the x-axis indicate the number in each maternal education stratum. P-value <0.0001, calculated from multinomial logistic regression model with 3-level outcome (un or under-vaccinated, added value from SIA, fully vaccinated prior to SIA) adjusted for age (years) at SIA and district. Right-hand figure is the percentage of the total survey population in each dose category, where the height of the bar and percentages listed above each bar reflects the percent on the population in that maternal education stratum. The percent of children in each dose category was calculated for each maternal education stratum, then multipled by the percent of children in that maternal education stratum.

The percentage of children who received their first or second dose from the SIA was highest among those whose mothers had only primary schooling or were illiterate compared to those with more educated mothers (Fig. 3A); in district-stratified analyses this trend was observed in all districts except Thiruvananthapuram District where over 90 % of mothers had middle level education or higher (Supplementary Table 7, Supplementary Fig. 4). Reaching all children with illiterate mothers through the SIA would result in a population-level added value of 4 % (Fig. 3B). The largest population-level added benefit of the SIA was among children from households with a mother of middle to higher secondary education (14 %) as most children in the survey were in this category (Fig. 3B). There was no association observed between sex and added value of the SIA in any district (Supplementary Table 7). For other characteristics, associations varied by district, with some evidence for increased value of the SIA among children living in households of non-permanent materials. In Palghar, Kanpur Nagar and Dibrugarh Districts the added value of the SIA was greatest in rural settings, while the percentage of children who remained unvaccinated or under-vaccinated following the SIA was largest among those living in urban slum settings.

## 4 Discussion

The added value of non-selective mass measles and rubella vaccination SIAs in settings with high routine immunization coverage has been questioned given their financial and personnel costs and potential inefficiency in reaching measles zero-dose and under-vaccinated children. We examined the added value of MR SIAs in reaching children unvaccinated and under-vaccinated against measles in five districts in India with moderate to high routine MCV1 and MCV2 coverage prior to the SIA. The relationship between routine and SIA coverage, and the routine vaccination status of those children vaccinated during the SIA, together determine the added value of the SIA in improving MCV coverage. This information can help to identify settings where non-selective SIAs (i.e., those that do not require verifying a child’s vaccination status), may be considered to reach measles zero-dose and under-vaccinated children and where more targeted, subnational vaccination approaches may be needed to identify and vaccinate these children [13], [22], [23].

In four of the five districts, 87.0 to 92.7 % of children had received at least one routine dose of MCV. Routine MCV1 coverage in Dibrugarh District was lower than expected (71.8 %), and lower than estimated using NFHS, however the children enrolled were older at the time of the survey than those used for the NFHS estimate (12–23 months). Although Thiruvananthapuram and Palghar Districts had high MCV1 coverage prior to the SIA (greater than 90 %), the net increase in coverage was higher in Palghar District where the SIA reached a larger percentage of the population and was more effective at reaching MCV zero-dose children. As a result, the percentage of children remaining zero-dose after the SIA was larger in Thiruvananthapuram relative to Palghar District, despite having similar routine MCV1 coverages, though not significantly different. In Dibrugarh District, only 71.8 % of children had received a routine dose of MCV prior to the SIA when “unknowns” were classed as unvaccinated. However, 94.9 % of zero-dose children were reached by the SIA, resulting in a 26.8 % net increase in coverage with at least one dose of MCV. Using this data, we estimated one zero-dose children was vaccinated for every-four children vaccinated during the SIA in Dibrugarh District, indicating substantial added value of the SIA. The performance of the SIAs overall and among zero-dose children varied greatly between districts and we did not find any consistent socio-demographic predictors that explained this variation.

The net increase in MCV2 coverage as a result of the SIA was considerably greater than that for MCV1 in four of the five sites, up to a 25 % increase in MCV2 coverage in Hoshiarpur District (routine MCV2 prior to SIA: 56.5 %). In the fifth site, Dibrugarh District, more children received added value in the form of a first dose relative to the second dose, and roughly half of children received additional benefit as a result of the SIA (Supplementary Table 6). Restricting to children above 24 months of age there was a slight decrease in the percent receiving their second dose through the SIA, although it still ranged from 4.1 % to 21.5 %. When treating unknown status as vaccinated the net increase in MCV2 was greater than that for MCV1 in Dibrugarh District, as observed in the other districts, since the net increase for MCV1 substantially decreased (from 26.8 % to 8.0 %). The percent of children receiving additional benefits as a result of the SIA decreased but remained the highest in Dibrugarh District after treating unknown status as vaccinated.

Approximately 50 to 70 % of children had received two routine doses and subsequently received the SIA dose. Often this is interpreted as unnecessary doses resulting in excess costs given that these children were fully vaccinated against measles prior to the SIA [22]. However, evidence of vaccination was based on parental recall for a considerable proportion of children in Kanpur Nagar and Palghar districts therefore reaching these children may have provided a missing dose that was incorrectly reported as having been received. Additionally, revaccinating these children may have potentially increased antibody levels among those where waning had occurred since the last dose, which in the short term can impact measles transmission and subsequently increase the impact of the SIA [24]. In the context of rubella vaccine introduction, most children received value from the supplementary dose, even if they had already received two doses of measles vaccine, as it provided the first opportunity for a rubella-containing vaccine [7]. The added value of the SIA in terms of rubella protection was also observed in our seroprevalence analysis [15].

The percentage of measles zero-dose children reached by the SIA in these districts ranged from 45.8 % to 94.9 %, substantially higher than that observed in 14 other countries using data from Demographic Health Surveys, in which only 1 % to 22 % of measles zero-dose children were reached by the SIA based on card documentation and recall [25]. In Jharkhand State in India, 6.3 % and 44.4 % of children aged 12 months to 5 years received their first or second dose of MCV during the 2010 measles vaccine SIA, respectively [26]. A post-coverage survey conducted in Liberia in 2018 estimated 78 % of zero-dose children received the SIA dose [12]. A similarly high proportion of previously unvaccinated children in Nigeria received a measles vaccine dose during the 2017–2018 measles SIA (82 %), higher than observed in most of the districts we studied [16]. However, routine MCV coverage in the Nigerian setting was estimated to be considerably lower (42 %) than in the five districts in India, resulting in a larger pool of zero-dose children to be vaccinated during the SIA.

The added value of the SIA was higher among children of less educated mothers and, in some districts, those living in rural settings or non-permanent structures, factors previously shown to be associated with lower routine coverage and highlight the SIA’s potential ability to reach children in these households [27], [28]. The ability of the SIA to reach these groups varied by district, however, with lower than expected results among zero-dose children in Kanpur Nagar (moderate routine coverage, primarily urban, moderate educational level), Hoshiarpur (moderate routine coverage, primarily rural, high educational levels) and Thiruvananthapuram districts (high routine coverage, primarily urban, very high educational level, relatively high use of private sector), highlighting the importance of local knowledge to inform targeted strategies to reach unvaccinated and under-vaccinated children [13]. It is also important to consider the balance between reaching groups with risk factors most likely to benefit from the SIA versus groups with weaker associations but who make up a larger proportion of the overall population, as observed with maternal education in this analysis.

Estimating vaccination coverage from surveys is challenging [29], [30]. Although routine immunization card availability was higher than in many DHS surveys [31], routine and SIA card availability varied by district, particularly SIA cards that may have been misplaced or were not provided to families due to operational issues. Due to lack of card availability, coverage estimates included both documented and recall doses. However, reported recall can be inaccurate [32], [33], [34], as observed in Dibrugarh District where 70 % of caregivers of children with documented MCV receipt were unaware of any MCV receipt when asked by recall. In the two districts with a higher degree of unknown MCV status (Hoshiarpur and Dibrugarh districts), awareness of MCV vaccination status was less common when the respondent was someone other than the mother.

The time between the SIA and survey was longer than recommended (typically within 3 months of the SIA), ranging from three months up to 16 months, which may have influenced availability of the card, recall of the SIA dose, and recall of routine doses as children participating in surveys that took place more than 10 months since the SIA were older than those in the surveys that took place sooner. To estimate impact of the SIA on routine coverage, we excluded doses documented to have been administered between the SIA and the survey. Although the date of supplementary dose receipt was recorded on the SIA card, we did not collect this date so we used the district-specific SIA start dates for all children. This may have excluded routine doses given before a child received the supplementary dose, especially for districts where the SIA occurred over a longer period than initially planned. For those children without routine cards, we assumed that recalled doses were given prior to the SIA, which may have overestimated routine coverage at the time of the SIA and underestimated the added value.

In this evaluation the key metric of interest was the percent of measles zero-dose children who were reached by the SIA. Due to the small number of children in this category prior to the SIA the precision of these estimates is limited, a common issue for many surveys conducted in settings with two MCV doses in the routine system and relatively high routine coverage [12]. We were also limited in our ability to describe characteristics of the children who remained measles zero-dose after the SIA, which were 20 children or fewer in each district. An additional limitation of the analysis is that we could not include children aged 5 to less than 15 years. We did not collect routine immunization data from these children due to concerns about availability of cards and quality of recall data for those older children without cards; some of that age cohort would also have been eligible for previous SIAs conducted in northern states in 2010–12 [35]. We anticipate the added value of the SIA on coverage may have been higher among the older children who would not have benefitted from intensified routine immunization activities in recent years and may therefore have been captured with a first or second dose through the SIA. This was also observed in our seroprevalence analyses where a gap in measles seropositivity prior to the SIA was filled following the SIA [15]. Depending on measles transmission in any given area, however, unvaccinated children may acquire measles before an SIA and reduce the added value from the SIA. Ideally, high quality data on coverage (by age cohort and strategy) will be combined with disease surveillance data to obtain better estimates of campaign efficacy [36].

Collecting and reporting on routine vaccination status prior to the SIA and receipt of the supplementary dose is important for understanding the correlation between doses and its impact on the effectiveness of the SIA. Collecting this information during the SIA minimizes issues of recall bias; however, this will be restricted to those who are successfully reached by the SIA and there are important logistical considerations such as impact on SIA activities [12]. Post-SIA surveys provide an opportunity to capture data for a broader sample, including those missed by routine and/or supplementary activities, but may be impacted by sampling and information biases. Regardless of the context used to collect the data, it is valuable to fully describe key metrics related to SIA effectiveness, such as the percentage of zero-dose children reached by the SIA, the percentage of fully vaccinated children reached by the SIA, and the percentage of children remaining zero-dose after the SIA (available from post-SIA surveys only). These metrics evaluating the added value of an SIA can support improved planning of targeted vaccination strategies to better reach children missed by routine or supplementary opportunities and may be used to compare different strategies across similar routine and SIA coverage settings [14].

As routine immunization improves, identifying and immunizing the few children who remain unvaccinated becomes increasingly difficult. In settings with high routine MCV coverage, non-selective SIAs will revaccinate a large proportion of children to reach measles zero-dose children. In the context of high routine coverage and suboptimal SIA coverage, few measles zero-dose children will be reached by the SIA. Targeted approaches, guided by local knowledge and innovative technologies and approaches, may be the preferred method for reaching measles zero-dose children [13], [37]. In settings with low MCV coverage, non-selective SIAs have greater potential to successfully reach measles zero-dose children, but need close evaluation to ensure that adequately high coverage is reached. In all settings, the ultimate indicator of success is sustained low or absent measles incidence, and greater investment in disease surveillance must accompany efforts to improve coverage measurement [38].

## CRediT authorship contribution statement

**C. Prosperi:** Conceptualization, Methodology, Investigation, Data curation, Writing – original draft, Writing – review & editing. **J.W.V. Thangaraj:** Conceptualization, Methodology, Investigation, Data curation, Writing – original draft, Writing – review & editing. **A.Z. Hasan:** Conceptualization, Methodology, Investigation, Data curation, Writing – original draft, Writing – review & editing. **M.S. Kumar:** Conceptualization, Methodology, Investigation, Data curation, Writing – original draft, Writing – review & editing. **S. Truelove:** Writing – review & editing. **V.S. Kumar:** Investigation, Data curation, Writing – review & editing. **A.K. Winter:** Writing – original draft, Writing – review & editing. **A.K. Bansal:** Investigation, Writing – review & editing. **S.L. Chauhan:** Investigation, Writing – review & editing. **G.S. Grover:** Investigation, Writing – review & editing. **A.K. Jain:** Investigation, Writing – review & editing. **R.N. Kulkarni:** Investigation, Writing – review & editing. **S.K. Sharma:** Investigation, Writing – review & editing. **B. Soman:** Investigation, Writing – review & editing. **I.K. Chaaithanya:** Investigation, Writing – review & editing. **S. Kharwal:** Investigation, Writing – review & editing. **S.K. Mishra:** Investigation, Writing – review & editing. **N.R. Salvi:** Investigation, Writing – review & editing. **N.P. Sharma:** Investigation, Writing – review & editing. **S. Sharma:** Investigation, Writing – review & editing. **A. Varghese:** Investigation, Writing – review & editing. **R. Sabarinathan:** Software, Investigation, Data curation, Writing – review & editing. **A. Duraiswamy:** Investigation, Writing – review & editing. **D.S. Rani:** Investigation, Writing – review & editing. **K. Kanagasabai:** Software, Investigation, Data curation, Writing – review & editing. **A. Lachyan:** Investigation, Writing – review & editing. **P. Gawali:** Investigation, Writing – review & editing. **M. Kapoor:** Investigation, Writing – review & editing. **S.K. Chonker:** Investigation, Writing – review & editing. **F.T. Cutts:** Writing – review & editing. **L. Sangal:** Conceptualization, Methodology, Writing – review & editing, Supervision, Project administration. **S.M. Mehendale:** Conceptualization, Methodology, Writing – review & editing. **G.N. Sapkal:** Conceptualization, Methodology, Writing – review & editing, Supervision, Project administration. **N. Gupta:** Conceptualization, Methodology, Investigation, Writing – review & editing, Supervision, Project administration. **K. Hayford:** Conceptualization, Methodology, Writing – original draft, Writing – review & editing, Funding acquisition, Supervision, Project administration. **W.J. Moss:** Conceptualization, Methodology, Writing – review & editing, Supervision, Project administration, Funding acquisition. **M.V. Murhekar:** Conceptualization, Methodology, Writing – review & editing, Supervision, Project administration.

## Declaration of Competing Interest

The authors declare that they have no known competing financial interests or personal relationships that could have appeared to influence the work reported in this paper.

## Data Availability

Data will be made available on request.
