# Supplementary material for: Added value of the measles-rubella supplementary immunization activity in reaching unvaccinated and under-vaccinated children, a cross-sectional study in five Indian districts, 2018–20
Source: Vaccine. 2023 Jan 9;41(2):486–95. doi: 10.1016/j.vaccine.2022.11.010 (PMC9831119; doi:10.1016/j.vaccine.2022.11.010)
Supplement: Supplementary data 1 [file mmc1.docx]

**Supplementary Methods**

**Study procedures**

After identifying the selected census enumeration block (CEB) and its boundaries, the team conducted a rapid mapping exercise to count the number of households and assessed if segmentation of the CEB was needed. A threshold of 70 households was set to ensure at least 13 individuals per age group were present in the study cluster. This threshold was based on birth rates, infant mortality ratio, and household size. If there were 70-140 households in the cluster, no segmentation was done. If there were more than 140 households, the cluster was segmented, and one segment was randomly selected by an independent statistician at ICMR-National Institute of Epidemiology, Chennai.

The survey team enumerated all individuals in the households in the study cluster, including those who slept in the houses in the prior night. A household was defined as a group of persons who normally live together and take their meals from a common kitchen unless the exigencies of work prevent any of them from doing so. Identification details of all individuals, including those who stayed in the house the previous night, including name, date of birth or age, gender, and availability for next 3 days, were collected using a tablet-based application. If the household was empty during enumeration the study team attempted to gather information about the household from a neighbor. If no information was available, the house was marked as ‘unavailable’ and any individuals living in that household were excluded from the sampling frame.

At the final stage, thirteen children were randomly selected from each of the two age strata using a tablet-based application, for a total of 26 children per cluster. Prior to enrollment, written informed consent was obtained from the parents/legal guardian and assent was obtained from children aged 7 to younger than 15 years. Up to three household visits were made to enroll selected individuals.

**Supplementary Figure 1. Map of India showing the states where the districts were selected for serosurveys conducted after the measles and rubella (MR) supplementary Immunization Activities, 2018-2020**


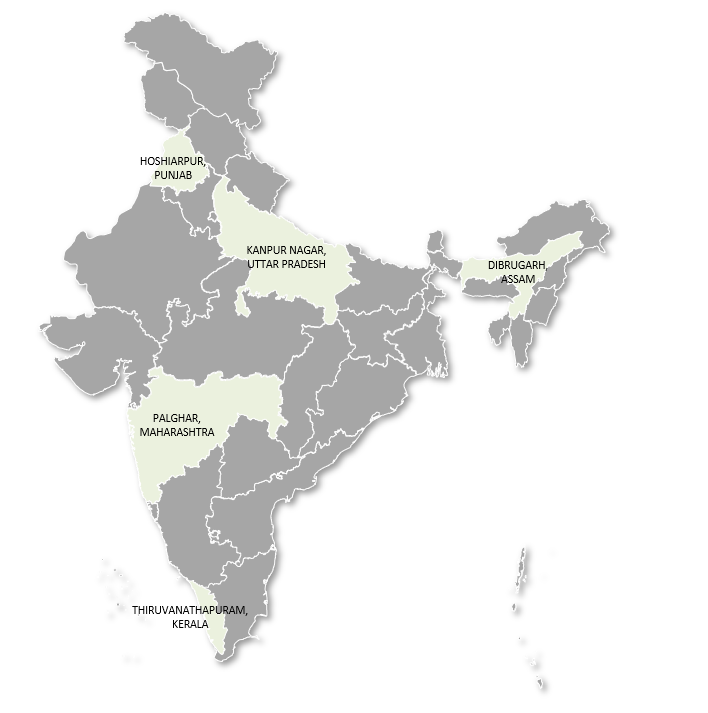


**Supplementary Figure 2: Flowchart describing the enrolment of participants in the post-SIA serosurveys by district**

**Thiruvananthapuram, Kerala**

**Household in the study clusters**

**Enumerated**

**Not Enumerated**

Households

3671 Households

2832 (77.1%) households

839 (22.9%) Households

782 Locked

3 Refused

54 Other

570

538 (94.4%)

387

Individuals

Enumerated

Available

Randomly Selected

340 (87.9%)

Enrolled

**Enumerated:** Visited all households in the cluster and collect identification details (name, date of birth/age, gender) **Available:** Children are available for the next three day**s Randomly selected:** Automated selection of children from enumeration data using android application. **Enrolled:** Data collected after obtaining consent/assent

**Dibrugarh, Assam**

2428 Households

1914 (78.8%) households

514 (21.2%) Households

427 Locked

19 Refused

68 Other

569

544 (95.6%)

%)

351

312 (88.9%)

**Hoshiarpur, Punjab**

3002 Households

2648 (88.2%) households

354 (11.8%) Households

278 Locked

45 Refused

31 Other

658

635 (96.5%)

390

346 (88.7%)

336 (86.8%)

387

724 (93.1%)

778

465 (14.1%) Households

310 Locked

126 Refused

29 Other

2832 (85.9%) households

3297 Households

**Palghar, Maharashtra**

**Kanpur Nagar, Uttar Pradesh**

2907 Households

2528 (87.0%) households

379 (13.0 %) Households

179 Locked

85 Refused

115 Other

819

702 (85.7%)

388

341 (87.9%)

**Supplementary Table 1. District-level characteristics**

|  | **Population (millions)^a^** | **Percent urban population^a^** | **Children 12-23 m** | | **Women 15-49 years** | |
| --- | --- | --- | --- | --- | --- | --- |
| **Site** |  |  | **MCV1 in 2019-2020, by card or recall^b^** | **Received most of vaccinations in private health facility^b^** | **Literacy rates^b^** | **10 or more years of schooling^b^** |
| Thiruvananthapuram District, Kerala | 3.3 | 53.7 | 93.4% | 14.3% | 98.5% | 83.1% |
| Kanpur Nagar District, Uttar Pradesh | 4.6 | 65.8% | 78.6% | 3.6% | 81.3% | 58.5% |
| Palghar District, Maharashtra | 2.8 | 49.2% | 100% | 0% | 77.6% | 48.3% |
| Hoshiarpur District, Punjab | 1.6 | 21.1% | 95.4% | 4.4% | 91.3% | 73.2% |
| Dibrugarh District, Assam | 1.3 | 18.4% | 97.8% | 7.6% | 76.6% | 33.7% |

a. Source: Government of India Census 2011.

b. National Family Health Surveys NFHS-5 (2019-2020).

**Supplementary Table 2. MCV parental recall among children aged 9 months to below 5 years of age at the time of the MR SIA with and without documented routine MCV among children with immunization card**

|  | **% with routine immunization card (95% CI)** | **Documented routine MCV1 or MCV2** | | **No documented routine MCV1 or MCV2** | |
| --- | --- | --- | --- | --- | --- |
| **Site (N)** |  | **n/N^a^** | **Sensitivity of parental recall^a^ (95% CI)** | **n/N^b^** | **False positive^b^ (95% CI)** |
| Thiruvananthapuram District, Kerala (N=340) | 84% (80, 87) | 256/273 | 94% (90, 96) | 7/8 | 88% (46, 98) |
| Kanpur Nagar District, Uttar Pradesh (N=341) | 52% (46, 57) | 154/157 | 98% (94, 99) | 9/16 | 56% (32, 78) |
| Palghar District, Maharashtra (N=336) | 67% (61, 72) | 173/192 | 90% (85, 94) | 11/  27 | 41% (24, 60) |
| Hoshiarpur District, Punjab (N=346) | 75% (70, 79) | 221/242 | 91% (87, 94) | 9/11 | 82% (49, 95) |
| Dibrugarh District, Assam (N=312) | 75% (70, 79) | 56/  214 | 26% (21, 32) | 5/19 | 26% (11, 50) |

a. Percent of children with card documented MCV1 or MCV2 where parent recalls MCV. n = number of children whose mothers reported MCV receipt; N = total number of children in that category of card documented vaccination

b. Percent of children with no card documented MCV1 and MCV2 where parent recalls MCV, either due to incorrect recall by the caregiver or issue with documentation at time of vaccination. n = number of children whose mothers reported MCV receipt; N = total number of children no card documented MCV1 and MCV2.

**Supplementary Table 3. Relationship of respondent to child by MCV data availability**

|  | **Hoshiarpur District, Punjab** | | | | **Dibrugarh District, Assam** | | | |
| --- | --- | --- | --- | --- | --- | --- | --- | --- |
|  | **N** | **MCV Known** | **MCV Unknown** | **N** | | **MCV Known** | **MCV Unknown** |  |
| **MCV1** |  |  |  |  | |  |  |  |
| Mother | 279 | 263 (94.3) | 16 (5.7) | 211 | | 178 (84.4) | 33 (15.6) |  |
| Other relative | 67 | 50 (74.6) | 17 (25.4) | 101 | | 68 (67.3) | 33 (32.7) |  |
| **MCV2** |  |  |  |  | |  |  |  |
| Mother | 279 | 241 (86.4) | 38 (13.6) | 211 | | 176 (83.4) | 35 (16.6) |  |
| Other relative | 67 | 34 (50.8) | 33 (49.2) | 101 | | 64 (63.4) | 37 (36.6) |  |

Row percentages. Chi-square p-value for all comparisons < 0.05.

**Supplementary Figure 3. Receipt of measles-containing vaccine among children 12 m to less than 5 years of age by site**


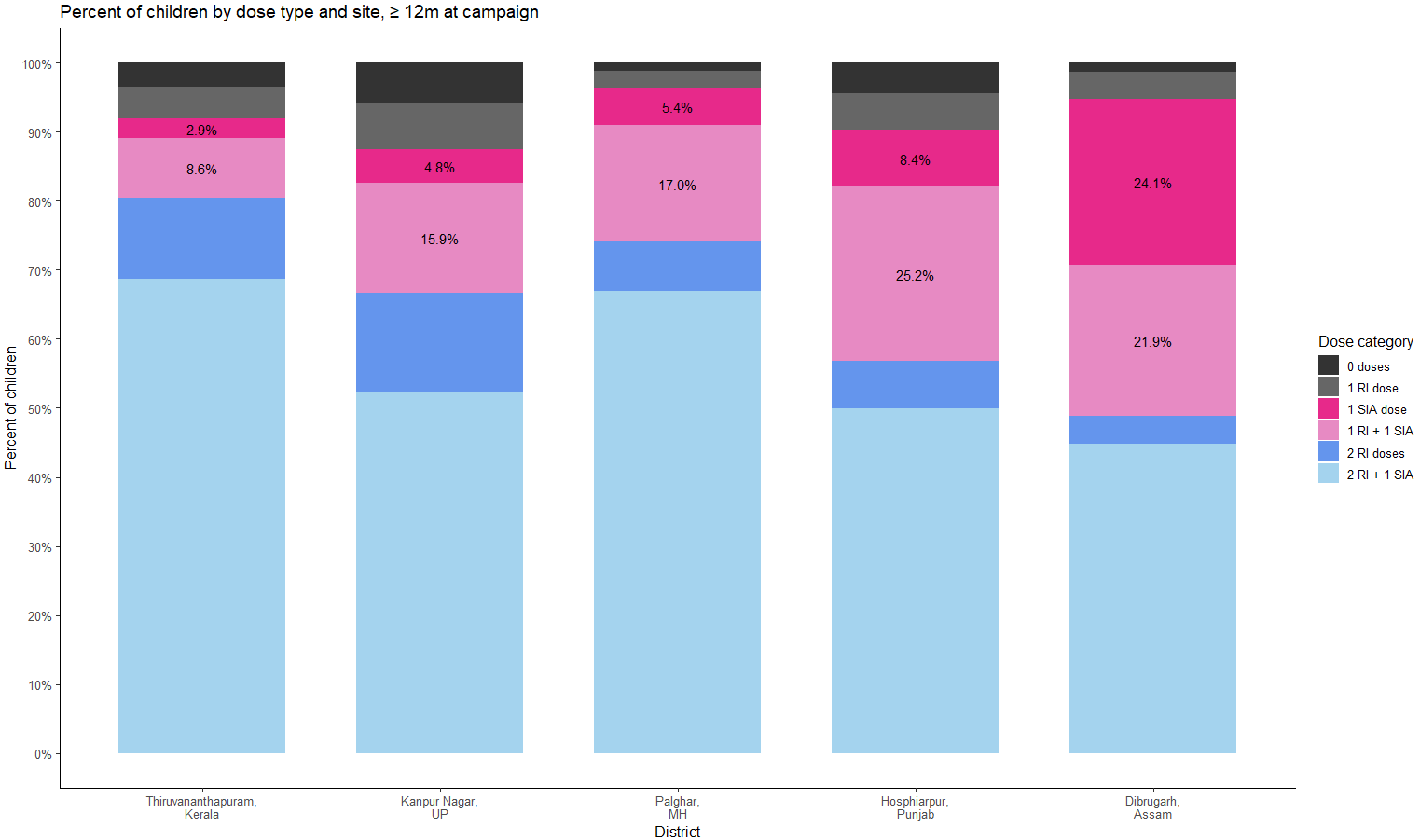


Bottom three bars reflect children who received at least one dose from the routine system. Pink bars reflect children who received added value from the SIA in terms of measles vaccine, either their first (dark pink) or second (light pink) dose of MCV. Dark gray bar at the top reflects the “zero MCV dose” children after SIA. Receipt for all doses is based on documented receipt plus recall. Percentages are survey weighted.

**Supplementary Figure 4. Receipt of measles-containing vaccine among children 24 m to less than 5 years of age by site**
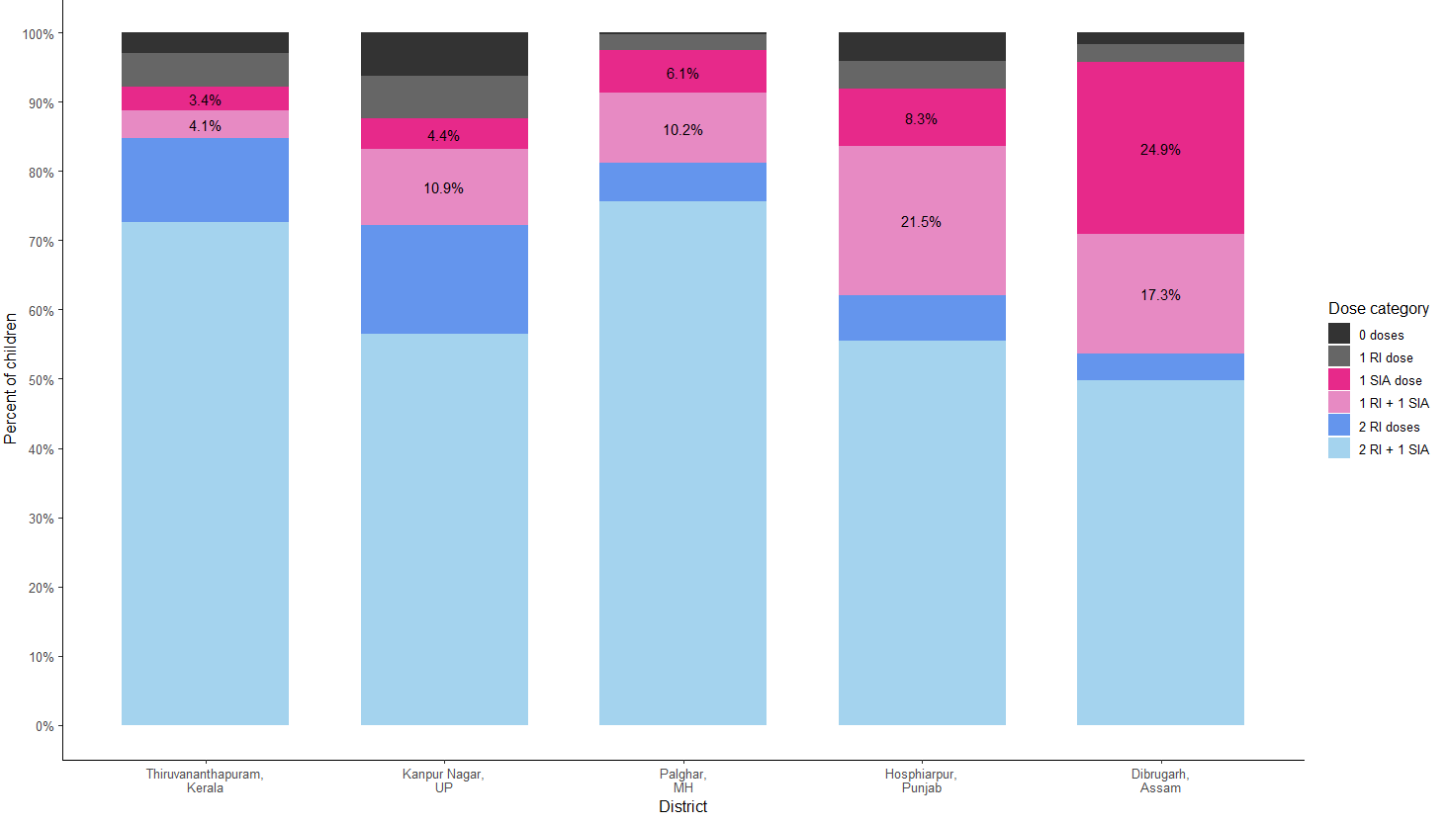


Bottom three bars reflect children who received at least one dose from the routine system. Pink bars reflect children who received added value from the SIA in terms of measles vaccine, either their first (dark pink) or second (light pink) dose of MCV. Dark gray bar at the top reflects the “zero MCV dose” children after SIA. Receipt for all doses is based on documented receipt plus recall. Percentages are survey weighted.

**Supplementary Table 4. Odds of SIA dose receipt among measles zero-dose children**

| **District** | **Odds ratio (95% CI)** | **Chi-sq p-value** |
| --- | --- | --- |
| Thiruvananthapuram District, Kerala | 0.70 (0.55, 0.88) | **0.002** |
| Kanpur Nagar District, Uttar Pradesh | 0.75 (0.63, 0.89) | **0.001** |
| Palghar District, Maharashtra | 0.97 (0.81, 1.16) | 0.72 |
| Hoshiarpur District, Punjab | 0.80 (0.68, 0.93) | **0.004** |
| Dibrugarh District, Assam | 1.07 (0.99, 1.14) | 0.09 |

Survey weighted logistic regression adjusted for age in years at time of campaign, with SIA dose receipt as the outcome and measles zero-dose prior to the SIA as the main predictor. Bold indicates chi-square p-value < 0.05.

**Supplementary Table 5a. Net increase in vaccination coverage as a result of the supplementary immunization activity (SIA), treating children with unknown vaccination status as unvaccinated**

|  | **Thiruvananthapuram Kerala** | | **Kanpur Nagar UP** | | **Palghar MH** | | **Hoshiarpur Punjab** | | **Dibrugarh Assam** | |
| --- | --- | --- | --- | --- | --- | --- | --- | --- | --- | --- |
| **At least 1 dose of MCV** | **N** | **%** | **N** | **%** | **N** | **%** | **N** | **%** | **N** | **%** |
| **Based on routine** | 305 | 93.4 | 299 | 87.8 | 321 | 92.6 | 297 | 87.0 | 222 | 71.8 |
| **+ SIA** | 10 | 3.0 | 20 | 5.8 | 22 | 6.4 | 28 | 8.2 | 83 | 26.8 |
| **2 doses of MCV** | **N** | **%** | **N** | **%** | **N** | **%** | **N** | **%** | **N** | **%** |
| **Based on routine** | 255 | 78.0 | 221 | 64.7 | 246 | 70.9 | 193 | 56.5 | 141 | 45.7 |
| **+ SIA** | 33 | 9.8 | 57 | 16.6 | 67 | 19.1 | 87 | 25.4 | 68 | 21.8 |
| **Received SIA dose** | 260 | 79.6 | 249 | 73.2 | 311 | 89.6 | 284 | 83.1 | 280 | 90.5 |
| **Remained zero dose after SIA** | 12 | 3.6 | 22 | 6.4 | 4 | 1.1 | 17 | 4.8 | 5 | 1.4 |
| **True zero-dose after SIA^a^** | 5 | 1.3 | 16 | 4.6 | 4 | 0.9 | 3 | 0.8 | 2 | 0.5 |
| **Unknown zero-dose after SIA ^a^** | 8 | 2.3 | 7 | 1.8 | 1 | 0.2 | 14 | 4.0 | 3 | 0.9 |
| **Ratio of zero dose or under-vaccinated children reached during the SIA relative to all reached by SIA^b^** | | | | | | | | | | |
| **Zero dose child:all children** | 1:27 | | 1:13 | | 1:15 | | 1:11 | | 1:4 | |
| **Under-vaccinated child: all children** | 1:9 | | 1:5 | | 1:5 | | 1:4 | | 1:5 | |

Restricted to children < 5 years. Receipt defined based on card plus recall. Weighted frequencies (rounded up to the whole number) and percentages for each dose category.

a. ‘True’ zero-dose defined as mother/caregiver reported child did not receive MCV and card is either available but blank for MCV or not available. Unknown zero-dose defined as no card available and mother/caregiver reported not knowing if child received MCV.

b. Ratio calculated as the weighted number receiving the SIA dose divided by the weighted number of children reached in the SIA who were previously zero dose or under-vaccinated (rounded up to the whole number). Children with unknown vaccination status were treated as unvaccinated.

**Supplementary Table 5b. Net increase in vaccination coverage as a result of the supplementary immunization activity (SIA), treating children with unknown vaccination status as vaccinated**

|  | **Thiruvananthapuram Kerala** | | **Kanpur Nagar UP** | | **Palghar MH** | | **Hoshiarpur Punjab** | | **Dibrugarh Assam** | |
| --- | --- | --- | --- | --- | --- | --- | --- | --- | --- | --- |
| **At least 1 dose of MCV** | **N** | **%** | **N** | **%** | **N** | **%** | **N** | **%** | **N** | **%** |
| **Based on routine** | 319 | 97.5 | 312 | 91.7 | 329 | 94.6 | 328 | 95.8 | 283 | 91.5 |
| **+ SIA** | 5 | 1.3 | 13 | 3.7 | 16 | 4.5 | 12 | 3.4 | 25 | 8.0 |
| **2 doses of MCV** | **N** | **%** | **N** | **%** | **N** | **%** | **N** | **%** | **N** | **%** |
| **Based on routine** | 270 | 82.4 | 236 | 69.2 | 261 | 75.2 | 266 | 77.7 | 209 | 67.6 |
| **+ SIA** | 35 | 10.6 | 57 | 16.6 | 59 | 17 | 53 | 15.5 | 63 | 20.3 |
| **Received SIA dose** | 274 | 83.8 | 254 | 74.7 | 312 | 89.8 | 291 | 85 | 283 | 91.5 |
| **Remained zero dose after SIA** | 5 | 1.3 | 16 | 4.6 | 4 | 0.9 | 3 | 0.8 | 2 | 0.5 |
| **Ratio of zero dose or under-vaccinated children reached during the SIA relative to all reached by SIA^a^** | | | | | | | | | | |
| **Zero dose child:all children** | 1:64 | | 1:21 | | 1:21 | | 1:26 | | 1:12 | |
| **Under-vaccinated child: all children** | 1:8 | | 1:5 | | 1:6 | | 1:6 | | 1:5 | |

Restricted to children < 5 years. Receipt defined based on card plus recall. Weighted frequencies (rounded up to the whole number) and percentages for each dose category.

a. Ratio calculated as the weighted number receiving the SIA dose divided by the weighted number of children reached in the SIA who were previously zero dose or under-vaccinated (rounded up to the whole number). Children with unknown vaccination status were treated as unvaccinated.

**Supplementary Table 6. Distribution of children by MCV dose category after the SIA, by site**

| **Site** | **Un or under-vaccinated^a^** | **Added value from SIA^b^** | **Fully vaccinated prior to SIA^c^** |
| --- | --- | --- | --- |
| **All children (treating unknown as unvaccinated)** | | | |
| Thiruvananthapuram District, Kerala | 9.1 | 12.8 | 78.0 |
| Kanpur Nagar District, Uttar Pradesh | 12.9 | 22.4 | 64.7 |
| Palghar District, Maharashtra | 3.7 | 25.4 | 70.9 |
| Hoshiarpur District, Punjab | 10.0 | 33.5 | 56.5 |
| Dibrugarh District, Assam | 5.7 | 48.6 | 45.7 |
| **All children (treating unknown as vaccinated)** | | | |
| Thiruvananthapuram District, Kerala | 5.8 | 11.9 | 82.3 |
| Kanpur Nagar District, Uttar Pradesh | 10.5 | 20.3 | 69.3 |
| Palghar District, Maharashtra | 3.3 | 21.4 | 75.3 |
| Hoshiarpur District, Punjab | 3.4 | 18.8 | 77.8 |
| Dibrugarh District, Assam | 4.0 | 28.4 | 67.6 |
| **After excluding children missing data for MCV1 or MCV2** | | | |
| Thiruvananthapuram District, Kerala | 7.2 | 11.3 | 81.5 |
| Kanpur Nagar District, Uttar Pradesh | 11.0 | 21.2 | 67.8 |
| Palghar District, Maharashtra | 3.4 | 22.4 | 74.1 |
| Hoshiarpur District, Punjab | 5.2 | 23.0 | 71.8 |
| Dibrugarh District, Assam | 5.2 | 36.1 | 58.7 |

SIA, supplementary immunization activity

a. Children with 0 or 1 MCV doses prior to the SIA and no SIA receipt.

b. Children receiving their first or second MCV dose from the SIA.

c. Children with two MCV doses prior to the SIA.

Survey weighted percentages.

**Supplementary Figure 5: Percent of the total survey population in each MCV dose category and maternal education stratum among children younger than 5 years of age at the time of the SIA, by district**


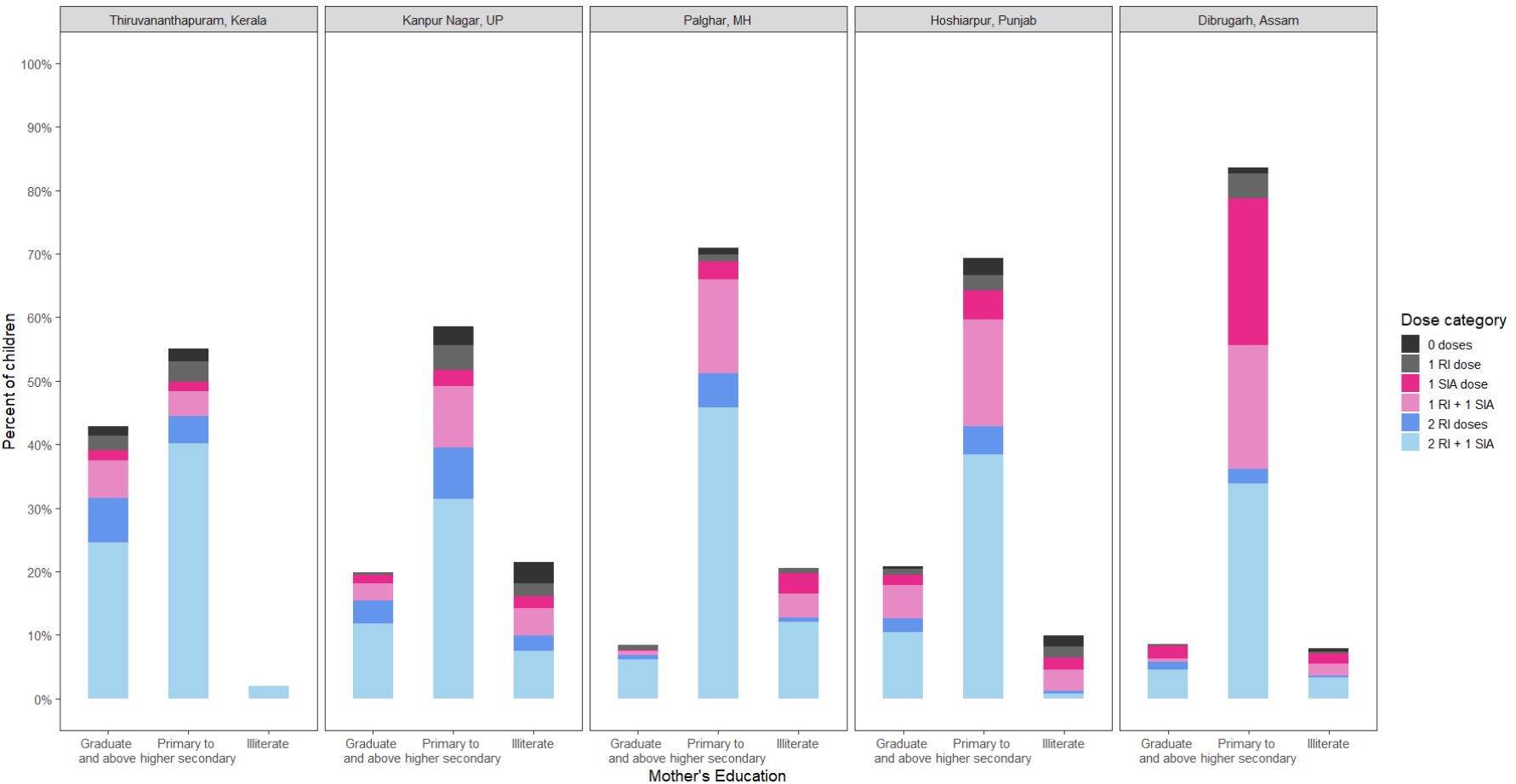


Percentage of the total survey population in each dose category, based on the distribution of the maternal education within each district. The survey weighted percent of children in each dose category was calculated for each maternal education stratum, then multipled by the survey weighted percent of children in that maternal educaion stratum.

**Supplementary Table 7. Association between added value of the supplemental immunization activity (SIA) and individual or household characteristics, by district**

|  | **Thiruvananthapuram District, Kerala** | | | **Kanpur Nagar District, Uttar Pradesh** | | | **Palghar District, Maharashtra** | | | | **Hoshiarpur District, Punjab** | | | | **Dibrugarh District, Assam** | | |
| --- | --- | --- | --- | --- | --- | --- | --- | --- | --- | --- | --- | --- | --- | --- | --- | --- | --- |
|  | **Un/under** | **Added** | **Fully** | **Un/under** | **Added** | **Fully** | **Un/under** | **Added** | **Fully** | **Un/under** | | **Added** | **Fully** | **Un/under** | | **Added** | **Fully** |
| Female | 14 (8.5) | 18 (10.9) | 133 (80.6) | 24 (14.5) | 27 (16.3) | 115 (69.3) | 8 (4.5) | 55 (30.9) | 115 (64.6) | 13 (7.6) | | 55 (32.2) | 103 (60.2) | 7 (4.7) | | 76 (51.4) | 65 (43.9) |
| Male | 12 (6.9) | 25 (14.3) | 138 (78.9) | 18 (10.3) | 40 (22.9) | 117 (66.9) | 4 (2.5) | 42 (26.6) | 112 (70.9) | 19 (10.9) | | 60 (34.3) | 96 (54.9) | 12 (7.3) | | 76 (46.3) | 76 (46.3) |
| **Maternal education level** | | | | | | | | | | | | | | | | | |
| Graduate or above | 10 (7.0) | 25 (17.6) | 107 (75.4) | **2 (2.4)** | **16 (19.5)** | **64 (78.0)** | 2 (4.1) | 7 (14.3) | 40 (81.6) | **5 (6.8)** | | **25 (33.8)** | **44 (59.5)** | **1 (4.3)** | | **8 (34.8)** | **14 (60.9)** |
| Middle to higher secondary | 13 (7.3) | 17 (9.5) | 149 (83.2) | **20 (12.8)** | **26 (16.7)** | **110 (70.5)** | 6 (3.4) | 49 (27.8) | 121 (68.8) | **12 (5.4)** | | **72 (32.1)** | **140 (62.5)** | **5 (3.4)** | | **68 (45.6)** | **76 (51.0)** |
| Primary | 3 (21.4) | 1 (7.1) | 10 (71.4) | **5 (10.9)** | **10 (21.7)** | **31 (67.4)** | 2 (5.4) | 12 (32.4) | 23 (62.2) | **5 (21.7)** | | **6 (26.1)** | **12 (52.2)** | **8 (7.1)** | | **64 (57.1)** | **40 (35.7)** |
| Illiterate | 0 (0.0) | 0 (0.0) | 4 (100) | **14 (25.5)** | **15 (27.3)** | **26 (47.3)** | 2 (2.7) | 29 (39.2) | 43 (58.1) | **10 (41.7)** | | **11 (45.8)** | **3 (12.5)** | **5 (20.8)** | | **10 (41.7)** | **9 (37.5)** |
| **Household materials** | | | | | | | | | | | | | | | | | |
| Permanent | 16 (7.2) | 27 (12.2) | 178 (80.5) | 19 (9.7) | 37 (19.0) | 139 (71.3) | **6 (4.0)** | **29 (19.5)** | **114 (76.5)** | **21 (6.9)** | | **98 (32.2)** | **185 (60.9)** | **3 (6.4)** | | **14 (29.8)** | **30 (63.8)** |
| Semi-permanent | 5 (5.8) | 12 (14.0) | 69 (80.2) | 16 (15.1) | 21 (19.8) | 69 (65.1) | **3 (2.5)** | **39 (32.2)** | **79 (65.3)** | **6 (28.6)** | | **7 (33.3)** | **8 (38.1)** | **3 (7.3)** | | **18 (43.9)** | **20 (48.8)** |
| Non-permanent | 5 (15.2) | 4 (12.1) | 24 (72.7) | 7 (17.5) | 9 (22.5) | 24 (60.0) | **3 (4.5)** | **29 (43.9)** | **34 (51.5)** | **3 (17.6)** | | **10 (58.8)** | **4 (23.5)** | **13 (5.8)** | | **120 (53.6)** | **91 (40.6)** |
| **Immunization facility type** | | | | | | | | | | | | | | | | | |
| Public | 21 (7.2) | 41 (14.1) | 228 (78.6) | 30 (10.2) | 60 (20.4) | 204 (69.4) | **10 (3.3)** | **95 (31.0)** | **201 (65.7)** | 26 (8.0) | | 110 (33.8) | 189 (58.2) | 17 (5.6) | | 149 (49.3) | 136 (45.0) |
| Non-public | 5 (10.2) | 2 (4.1) | 42 (85.7) | 5 (13.2) | 6 (15.8) | 27 (71.1) | **2 (6.7)** | **2 (6.7)** | **26 (86.7)** | 3 (18.8) | | 5 (31.3) | 8 (50.0) | 2 (20.0) | | 3 (30.0) | 5 (50.0) |
| **Setting** | | | | | | | | | | | | | | | | | |
| Urban non-slum | 14 (12.8) | 11 (10.1) | 84 (77.1) | **6 (6.5)** | **12 (13.0)** | **74 (80.4)** | **3 (3.3)** | **17 (18.7)** | **71 (78.0)** | 8 (7.4) | | 34 (31.5) | 66 (61.1) | **3 (8.1)** | | **10 (27.0)** | **24 (64.9)** |
| Urban slum | 1 (1.8) | 9 (16.1) | 46 (82.1) | **28 (21.5)** | **29 (22.3)** | **73 (56.2)** | **6 (7.3)** | **17 (20.7)** | **59 (72.0)** | 1 (8.3) | | 5 (41.7) | 6 (50.0) | **5 (16.7)** | | **10 (33.3)** | **15 (50.0)** |
| Rural | 11 (6.3) | 23 (13.1) | 141 (80.6) | **8 (6.7)** | **26 (21.8)** | **85 (71.4)** | **3 (1.8)** | **63 (38.7)** | **97 (59.5)** | 23 (10.2) | | 76 (33.8) | 126 (56.0) | **11 (4.5)** | | **132 (53.9)** | **102 (41.6)** |

‘Un/under’ indicates the child had received zero or one dose prior to the SIA and did not receive the SIA. ‘Added’ indicates the child received either their first or second dose from the SIA. ‘Fully’ indicates the child was fully vacinated prior to the SIA (regardless of SIA receipt). Bold indicates p-value < 0.1, where p-values calculated from site-specific glm models with 3-level outcome (un or under-vaccinated, added value from SIA, fully vaccinated prior to SIA) adjusted for age (years) at SIA.
